# Effects of adding acupuncture to group psychotherapy for anxiety

**DOI:** 10.1192/j.eurpsy.2024.883

**Published:** 2024-08-27

**Authors:** L. Mehl-Madrona

**Affiliations:** Native Studies, University of Maine, Orono, United States

## Abstract

**Introduction:**

Acupuncture has long been used in treating anxiety, and a literature exists on its effectiveness. However, acupuncture is rarely covered by government insurance (Medicaid or Medicare) or even by many commercial insurance carriers in the United States, making it inaccessible to those who cannot pay separately.

**Objectives:**

We asked if adding acupuncture to an anxiety group would improve outcome.

**Methods:**

We provided acupuncture during group psychotherapy for anxiety as a non-billable service. This was feasible since patients were already being billed for group psychotherapy. A physician and a social work intern led the group. At the start of the group, the physician went around the circle of group members and inserted acupuncture needles, using points in the ears, head, hands, feet, and, in the summer, arms and lower legs). The size of the group ranged from 4 to 12 people. We used Battlefield auricular points, the four gates (Large Intestine 4 and Liver 3, bilaterally), and GV24, GV29, Ht7, and Sp6. Sometimes, other points were added for other symptoms (back pain, neck pain, etc.) People sometimes joined the group without anxiety as a core problem in getting access to acupuncture. A core group of patients formed who came weekly while others came and went. The Hamilton Anxiety Scale measured anxiety after treatments 4, 8, and 12. The group lasted 90 minutes and consisted of mindfulness training, guided imagery, and CBT for anxiety. All patients met the criteria for generalized anxiety disorder. The t-test procedure was used to compare the differences between the means for the two groups.

**Results:**

Thirty-five patients received acupuncture, while another 55 patients attended the group and did not elect to receive acupuncture. All patients were covered by MaineCare health insurance, Maine’s version of Medicaid. All patients had multiple other medical problems, which was why they were referred to the group. Seventy percent of the patients were women, and 30% were men. The average age was 40.1 years. Anxiety ratings on the Hamilton Anxiety Scale decreased by the last time measured for those not receiving acupuncture by an average of 5.17 points (S.D. 2.9; n = 55). Anxiety ratings for those receiving acupuncture decreased by an average of 7.19 points (S.D. 2.5, n = 35). The difference of the means was -2.02 (S.E. 0.595; 95% CI = -2.203 to -0.837; t = -3.394; p = 0.001). Headaches, shoulder pains, and upper back pain also decreased. Patients reported high levels of benefit from the acupuncture and encouraged other patients to continue to come and try the acupuncture. Usually, the needles could be placed within the first third of the group.

**Conclusions:**

Acupuncture improved anxiety ratings for people in group psychotherapy for anxiety over group alone, though the possibility of a placebo effect cannot be eliminated. Patients chose acupuncture, which could also present a potential bias.

**Disclosure of Interest:**

None Declared

